# Impact of Vitamin C and Thiamine Administration on Delirium-Free Days in Patients with Septic Shock

**DOI:** 10.3390/jcm9010193

**Published:** 2020-01-10

**Authors:** Jong Eun Park, Tae Gun Shin, Ik Joon Jo, Kyeongman Jeon, Gee Young Suh, Minsu Park, Hojeong Won, Chi Ryang Chung, Sung Yeon Hwang

**Affiliations:** 1 Department of Emergency Medicine, Samsung Medical Center, Sungkyunkwan University School of Medicine, Seoul 06351, Korea; jongeun7.park@samsung.com (J.E.P.); taegunshin@skku.edu (T.G.S.); ikjoon.jo@samsung.com (I.J.J.); 2 Department of Critical Care Medicine, Samsung Medical Center, Sungkyunkwan University School of Medicine, Seoul 06351, Korea; kyeongman.jeon@samsung.com (K.J.); gy.suh@samsung.com (G.Y.S.); 3 Division of Pulmonary and Critical Care Medicine, Department of Medicine, Samsung Medical Center, Sungkyunkwan University School of Medicine, Seoul 06351, Korea; 4 Statistics and Data Center, Samsung Medical Center, Seoul 06351, Korea; minsu51.park@samsung.com (M.P.); hojeong.won@sbri.co.kr (H.W.)

**Keywords:** septic shock, sepsis, delirium, vitamin C, thiamine

## Abstract

Sepsis is a common cause of delirium in the intensive care unit (ICU). Recently, vitamin C and thiamine administration has been gaining interest as a potential adjunct therapy for sepsis. We investigated the impact of early vitamin C and thiamine administration on ICU delirium-free days among critically ill patients in septic shock. We performed a single-center, retrospective study of patients who visited the emergency department (ED) from January 2017 to July 2018. We categorized patients into a treatment (received vitamin C and thiamine) and control group. We compared delirium-free days within 14 days after ICU admission using propensity score matching. Of 435 patients with septic shock, we assigned 89 propensity score-matched pairs to the treatment and control groups. The median delirium-free days did not differ between treatment (11, interquartile range [IQR] 5–14 days) and control (12, IQR 6–14 days) groups (*p* = 0.894). Secondary outcomes were not different between the two groups, including delirium incidence and 28-day mortality. These findings were consistent after subgroup analysis for patients who met the sepsis-3 definition of septic shock. Vitamin C and thiamine administration showed no association with ICU delirium-free days among patients in septic shock.

## 1. Introduction

Delirium is an acute brain dysfunction that is characterized by a disturbance in attention and awareness, cognitive impairment, and psychomotor behavioral disturbances that develop over a short period and fluctuates during the day [1]. Delirium commonly occurs in critically ill patients with a prevalence as high as 77% in the intensive care unit (ICU) [2,3]. Delirium in the ICU is associated with increased mortality, longer hospital stay, higher medical cost, and long-term cognitive impairment [3,4,5]. Therefore, efforts to properly manage delirium are important to prevent and decrease its negative consequences.

Both vitamin C and thiamine deficiencies are common in septic patients [6,7]. Evidence suggests that these vitamin deficiencies may have implications in the pathogenesis of delirium [8,9]. Vitamin C plays important roles, including the scavenging of free radicals, improvement of the endothelial and blood–brain barrier functions, and the modulation of neurotransmitters, which are all involved in the pathophysiology of delirium [10,11]. Clinically, vitamin C deficiency (scurvy) manifests with the signs and symptoms of impaired neuropsychiatric function including apathy, irritability, and psychomotor retardation [12]. An observational study performed in a geriatric population showed that patients with delirium had significantly lower serum vitamin C concentrations than patients without delirium [13]. Thiamine may also play roles in the neuroprotection and balancing of neurotransmitters [14,15]. Thiamine deficiency can lead to decreased production of adenosine triphosphate (ATP), since it plays a role as a cofactor in the Kreb’s cycle. Decreased ATP production in the brain leads to an increase in the dopamine concentration, which may lead to a delirious state. Thiamine is also essential for the synthesis of neurotransmitters such as acetylcholine and gamma aminobutyric acid, and a decrease in the level of these neurotransmitters has been linked to delirium [9]. Thiamine is involved in the glutathione-producing pathway as a cofactor. Decrease in glutathione leads to an impairment in the scavenging of free radicals that causes cellular injuries in the neuronal cells, thus further contributing to the development of delirium [9].

Recent clinical studies showed potentially therapeutic benefits of vitamin C and thiamine on various clinical outcomes in septic patients [16,17,18,19]. Given that pre-existing evidence showed vitamin C and thiamine deficiencies to be common in septic patients and that this may play a role in the development of delirium, these vitamins may be helpful adjuncts in the management of delirium in septic patients. However, the effect of vitamin supplementation on sepsis-associated delirium has been poorly investigated. The objective of this study was to evaluate the impact of early combination therapy with vitamin C and thiamine on ICU delirium-free days in patients with septic shock who were admitted via the emergency department (ED).

## 2. Materials and Methods

### 2.1. Study Design and Population

This was a single-center, retrospective study involving patients with septic shock who visited the ED at the Samsung Medical Centre (a 1960-bed, university-affiliated, tertiary care referral hospital located in a metropolitan city with an annual census of over 70,000 people). The study period was January 2017 through July 2018. The Institutional Review Board of Samsung Medical Centre (IRB file number, 2018-11-015) approved this study. Need for informed consent was waived because this study was retrospective, observational, and the patient data were anonymized.

The inclusion criteria were as follows: (1) ≥18 years; (2) diagnosed with septic shock during their ED stay and admitted to the ICU from the ED. Patients who were transferred to the general ward, signed a “do not attempt resuscitation order,” were discharged to home, or expired in the ED were excluded from the analysis.

### 2.2. Vitamin Administration and Patient Management

Vitamin C and thiamine administration was adopted as an adjunct therapy for septic shock after July 2017. Vitamin C (3 g/12 h or 1.5 g/6 h) and thiamine (200 mg/12 h) mixed in 50- or 100-mL solution bags of 5% dextrose in water or normal saline were administered intravenously within 6 h of shock recognition. The decision to administer vitamins was at the discretion of the treating physician in the ED and ICU. Protocol-driven therapies based on the updated Surviving Sepsis Campaign guidelines were provided for all included patients [20]. We categorized the study population into treatment (received vitamin C and thiamine) and control groups.

### 2.3. Definition and Data Collection

Septic shock was defined as refractory hypotension requiring vasopressors to maintain the mean arterial pressure (MAP) at ≥65 mmHg despite adequate fluid therapy (at least 1 L or 20–30 mL/kg of crystalloid solution administered over 30 min), or hypoperfusion (defined as a serum lactate concentration ≥4 mmol/L) in patients with suspected or confirmed infection [21].

The Richmond Agitation Sedation Scale (RASS) and the validated Korean version of the Confusion Assessment Method for the ICU (CAM-ICU) were used to assess all patients in the ICU at least once per nursing shift during the study period [22,23]. If the RASS score was −3 or higher and the CAM-ICU was positive at least once within 24 h, the patient was considered to have delirium. If the RASS score was −4 or lower, the patient was considered to be comatose and CAM-ICU was not assessed in that case. Delirium-free days were defined as the number of days during which the patient was alive without delirium within the first 14 days after ICU admission. Delirious patients who died were considered to have zero delirium-free days. Delirium-coma-free days were defined as the number of days within the first 14 days after ICU admission during which the patient was alive without delirium or coma. Patients who died while delirious or comatose were considered to have zero delirium coma-free days.

The following data were extracted from the prospectively collected registry and electronic medical records: general patient characteristics, including age, sex, and comorbidities; infection focus based on the final diagnosis at hospital discharge; mechanical ventilation; laboratory tests; hospital and ICU length of stay (LOS); RASS score; CAM-ICU, and survival data. If a patient was discharged before the 28th day of hospitalization, we used the visit history after discharge, the mortality data provided by Statistics Korea, and telephone interviews to collect the data. Sequential Organ Failure Assessment (SOFA) scores and Acute Physiology and Chronic Health Evaluation (APACHE) II scores were calculated on the day of admission.

### 2.4. Outcome Measures

The primary outcome was delirium-free days. The secondary outcomes were delirium incidence within 14 days, delirium-coma-free days, duration of delirium, hospital and ICU LOS, and 28-day mortality.

### 2.5. Primary Data Analysis

We present the data as means ± standard deviations or medians with interquartile ranges (IQRs) for continuous data, and numbers with percentages for categorical data. Continuous variables were compared using the Student’s *t*-test or the Wilcoxon rank-sum test, while categorical variables were compared using the chi-squared test or Fisher’s exact test. We used propensity score matching (PSM) to adjust for patient imbalance between the treatment and control groups using variables including age, the sepsis-3 definition of septic shock, lactate level at the time of presentation, infection focus, creatinine, albumin, steroid use, the maximum SOFA score within 24 h, APACHE II score, the use of mechanical ventilation, benzodiazepine use, and initial presentation of delirium at the time of ICU admission. We performed 1-to-1 nearest-neighbor matching with caliper = 0.1 and the covariate balance between the treatment and control groups was evaluated based on the standardized mean differences. Among the original study population, we made an additional PSM set for a subgroup of patients who met the 2016 sepsis-3 definition of septic shock (defined as persistent hypotension requiring vasopressors to maintain MAP at ≥65 mmHg and a serum lactate level >2 mmol/L despite adequate volume resuscitation) [24]. Differences between the matched pairs were compared using the Wilcoxon signed rank test or paired Student’s *t*-test for continuous variables and the McNemar’s test for categorical variables. All two-tailed *p*-values <0.05 were considered statistically significant. The Cohen’s d procedure was applied to calculate the effect size, and categorized using the standardized effect size thresholds: <0.1 no, 0.1–0.4 small, 0.5–0.7 medium, and >0.8 large [25,26]. The statistical analysis was executed using SAS version 9.4 (SAS Institute, Cary, North Carolina, USA), R 3.5.2 (The R Foundation, Vienna, Austria; http://www.r-project.org/), and STATA 15.0 (STATA Corporation, College Station, Texas, USA) by independent biostatisticians.

## 3. Results

### 3.1. Baseline Characteristics

**Figure 1:**
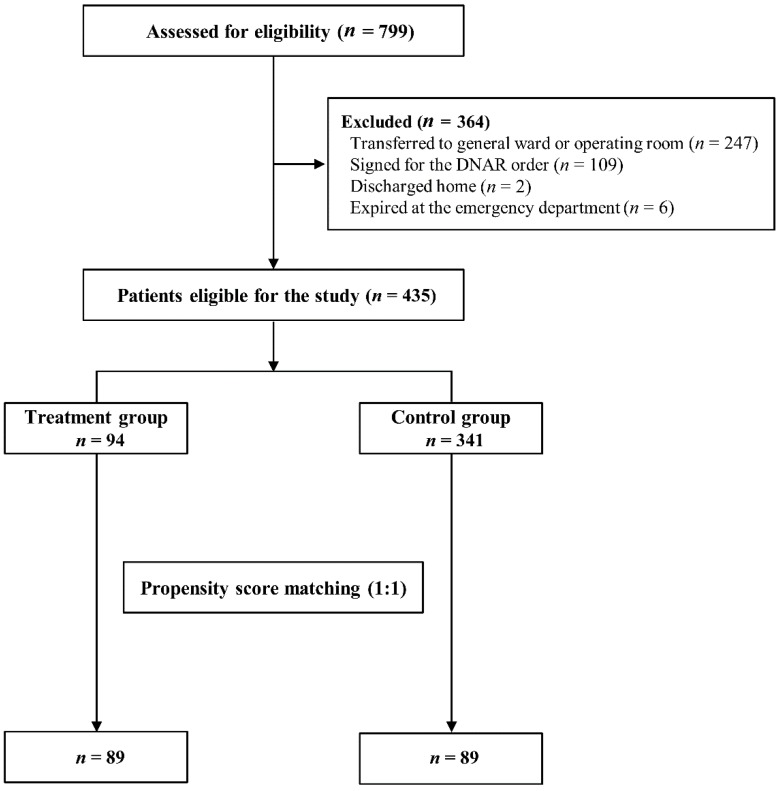
Study flowchart. DNAR, do not attempt resuscitation.

A total of 799 patients were diagnosed with septic shock during the study period. Of these, 364 were excluded, and the remaining 435 patients were analyzed (Figure 1). Of the eligible patients, we assigned 94 (21.6%) to the treatment group and 341 (78.4%) to the control group. The median duration of vitamin C and thiamine administration was 2 (IQR, 1–4) days. The baseline characteristics of the overall population and propensity score-matched patients are shown in Table 1. Age and sex were not significantly different between the two groups in the unmatched population. APACHE II score (30.2 ± 8.1 vs. 28.4 ± 9.2, *p* = 0.044), SOFA score (11.5 ± 3.4 vs. 10.1 ± 3.7, *p* < 0.001) and serum lactate level (5.5 ± 3.1 vs. 4.6 ± 3.2, *p* = 0.003) were significantly higher in the treatment group in the unmatched population. Among the 435 patients, 89 propensity score-matched pairs were generated after 1-to-1 PSM. The PSM method resulted in balanced groups in terms of the baseline characteristics.

Of the total 435 study participants, 295 met the criteria for the sepsis-3 definition of septic shock. Of the eligible patients, we assigned 80 (27.1%) to the treatment group and 215 (72.9%) to the control group. An additional 1-to-1 PSM was performed on these patients, resulting in 73 propensity score-matched pairs. The baseline characteristics of the subgroup patients who met the sepsis-3 definition of septic shock are presented elsewhere (see Supplement, Table S1). The PSM method resulted in balanced groups in terms of the baseline characteristics in this subgroup population.

### 3.2. Outcomes

Before PSM, the median delirium-free days was significantly shorter in the treatment group than in the control group (11 [IQR 4–14] vs. 13 [IQR, 7–14] days, respectively, *p* = 0.042, Cohen’s d = 0.188) (Table 2). However, it was not different between the treatment group and the control group after PSM (11 [IQR 5–14] vs. 12 [IQR 6–14] days, respectively, *p* = 0.894, Cohen’s d = 0.035). The secondary outcomes including the delirium coma-free days, the delirium incidence, the duration of delirium, the hospital LOS, the ICU LOS, and the 28-day mortality were also not different between the two groups after PSM.

The comparison of the outcomes in the subgroup of patients who met the sepsis-3 definition of septic shock is presented elsewhere (see Supplement, Table S2). The median delirium-free days was not significantly different between the treatment group and the control group (11 [IQR 4–14] vs. 11 [IQR 2–14] days, respectively, *p* = 0.531, Cohen’s d = 0.109) in the subgroup after PSM.

## 4. Discussion

The pathophysiology of delirium appears to be multifactorial. Potential mechanisms may include impaired oxidative metabolism, thrombosis, microvascular damage, inflammatory cytokines, structural and functional alterations of the blood–brain barrier, impaired cerebral microcirculatory blood flow, and altered neurotransmitter balance [11,27,28]. Vitamin C and thiamine are potential candidates to reduce these pathophysiologic processes by scavenging on the free radicals, decreasing endothelial injury and inflammation, supporting cytokine metabolism, maintaining the neurotransmitter balance, and preventing neuronal damage. Despite the theoretical benefits, our results showed that vitamin C and thiamine administration was not associated with increased delirium-free days in septic shock patients who presented at the ED. To our knowledge, this is the first study to investigate whether the combination therapy of vitamin C and thiamine could improve the outcomes associated with delirium in the ICU.

There are several possible explanations for why vitamin C and thiamine administration did not affect delirium-free days in this study. First, numerous factors are involved in the occurrence and maintenance of delirium. For instance, psychoactive drugs may disturb neurotransmission in the brain, provoking a delirious state [29]. Thus, the benefits of vitamin C and thiamine administration may have been less effective in these cases. Similarly, given the complex pathophysiology of septic shock, the administration of only two vitamins might be insufficient. Secondly, the dose and duration of vitamin C and thiamine administration might have been insufficient. Patients were treated with 6 g of vitamin C and 400 mg of thiamine per day, with a median duration of 2 (IQR, 1–4) days. Although the vitamins were administered to the patients based on previous studies, the optimal dose and duration of vitamin C and thiamine administration in patients with septic shock have not been established. Thirdly, serum vitamin C and thiamine level measurements were not performed. In a study of the impact of vitamin D supplementation on outcomes in critically ill patients with vitamin D deficiency, administration of high-dose vitamin D3 reduced hospital mortality only in the subgroup with severe vitamin D deficiency [30]. If the vitamin deficiencies in the patients included in our study were not severe, then vitamin C and thiamine administration would have been unlikely to produce significant effects. Fourth, Marik et al. [31] administered vitamin C, thiamine, and steroid cocktails to septic patients. They argued that this method had a synergistic effect. We evaluated only the combination of vitamin C and thiamine in this study. However, steroids were administered in most patients according to the Surviving Sepsis Campaign guidelines and we adjusted for the administration of steroids using PSM.

Previous studies have demonstrated that vitamin C or thiamine deficiencies might be relevant in acute brain dysfunction. Torbergsen et al. [13] conducted a case-control study to assess the association between hypovitaminosis and delirium in hip fracture patients. They found that the concentration of vitamin C was significantly lower in patients with delirium than in those without delirium. Voigt et al. [10] measured vitamin C concentration in patients with septic encephalopathy. They demonstrated that the vitamin C concentration was significantly lower in both the plasma and cerebrospinal fluid in patients with septic encephalopathy than in controls, and the degree of decrease in vitamin C in the cerebrospinal fluid correlated with the severity of the neurological symptoms. O’Keeffe et al. [32] measured thiamine levels in geriatric patients who were admitted to hospital. They reported that delirium occurred more frequently in thiamine-deficient patients than in patients without this deficiency (76% vs. 32%, *p* < 0.025).

Some previous studies have shown that vitamin C has a potential therapeutic effect on mental illness. In a randomized controlled trial of acutely hospitalized patients, Zhang et al. [33] demonstrated that vitamin C (500 mg per oral, twice daily) improved the mood and decreased depression symptoms. In randomized double-blind trials, Brody et al. [34] showed that oral vitamin C decreased the Beck Depression scores and Amr et al. [35] showed that vitamin C had an effect, in addition to fluoxetine, in the treatment of major depression in pediatric patients. Vitamin C may have a role in relieving acute and chronic pain in various groups [36]. There is, however, no definite clinical evidence to show the effect of vitamin C administration on delirium. We need further clinical trials focusing on delirium.

Recent studies have reported promising outcomes (prevention of progressive organ dysfunction and reduced mortality) of vitamin C and thiamine treatment in patients with septic shock [31,37]. However, findings on the effect of vitamin C and thiamine are still controversial [38,39]. Of note, there are ongoing clinical trials to determine the efficacy of vitamin C and thiamine therapy in septic shock. In one of these studies, delirium is being assessed as a secondary outcome [40]. This study may provide clearer insights into the roles of these vitamins in delirium management in septic patients.

This study has some limitations. First, this study has inherent limitations as a retrospective observational study. In particular, the vitamin treatment was not randomized and the decision to initiate and the timing of discontinuation of the vitamins were at the discretion of the treating physician in the ED and ICU. Secondly, the study was conducted in the ED and ICU of an academic hospital, thus limiting the generalization of the results of this study to other settings. Thirdly, the administration of vitamin C and thiamine to patients with septic shock was not protocolized at the beginning of the study at our institution, but was only adopted during the study periods. Thus, the vitamins were not administered to the included patients until the adoption of this therapy.

## 5. Conclusions

Early administration of vitamin C and thiamine therapy did not increase the number of delirium-free days in patients with septic shock in this study. The combination therapy had no clinical impact on other outcomes including delirium coma-free days, delirium incidence, duration, hospital and ICU LOS, and 28-day mortality. Further prospective trials are necessary to clarify the clinical roles of vitamin C and thiamine therapy in delirium in septic patients.

## Figures and Tables

**Table 1:** Baseline characteristics of the overall and matched cohorts.

Variables	Before Matching			After Matching		
	Treatment (*n* = 94)	Control (*n* = 341)	*p*	Treatment (*n* = 89)	Control (*n* = 89)	*p*
Age, years	69 (60–76)	69 (61–76)	0.943	69 (61–76)	71 (62–78)	0.653
Sex, male	55 (58.5)	229 (67.1)	0.119	52 (58.4)	55 (61.8)	0.662
Comorbidities						
Diabetes	32 (34)	117 (34.3)	0.961	31 (34.8)	29 (32.6)	0.768
Hypertension	36 (38.3)	139 (40.8)	0.666	34 (38.2)	35 (39.3)	0.884
Cardiac disease	11 (11.7)	62 (18.2)	0.137	11 (12.4)	16 (18)	0.251
Cerebral vascular disease	9 (9.6)	27 (7.9)	0.606	9 (10.1)	8 (9)	0.782
Chronic lung disease	8 (8.5)	36 (10.6)	0.560	8 (9)	9 (10.1)	0.808
Chronic renal disease	7 (7.5)	23 (10.3)	0.413	7 (7.8)	9 (10.1)	0.593
Chronic liver disease	15 (16)	47 (14)	0.593	15 (16.9)	19 (21.4)	0.465
Hematologic malignancy	13 (13.8)	32 (9.4)	0.210	12 (13.5)	10 (11.2)	0.655
Metastatic cancer	20 (21.3)	92 (27)	0.263	19 (21.4)	17 (19.1)	0.706
Infection focus			0.006			>0.999
Respiratory	24 (25.5)	140 (41.1)		23 (25.8)	23 (25.8)	
Non-respiratory	70 (74.5)	201 (58.9)		66 (74.2)	132 (74.2)	
APACHE II score	30.2 ± 8.1	28.4 ± 9.2	0.044	30.0 ± 7.9	30.0 ± 8.9	0.697
SOFA score	11.5 ± 3.4	10.1 ± 3.7	<0.001	11.4 ± 3.5	11.5 ± 3.4	0.854
Sepsis-3 definition *	80 (85.1)	215 (63.1)	<0.001	75 (84.3)	73 (82)	0.593
Laboratory tests						
Lactate (mmol/L)	5.5 ± 3.1	4.6 ± 3.2	0.003	5.2 ± 2.7	4.8 ± 2.9	0.431
Albumin (mg/dL)	3.1 ± 0.6	3.2 ± 0.6	0.209	3.1 ± 0.6	3.1 ± 0.6	0.742
Creatinine (mg/dL)	2.1 ± 1.5	1.8 ± 1.6	0.019	1.9 ± 1.4	2.2 ± 2.3	0.934
Mechanical ventilation use †	53 (56.4)	173 (50.7)	0.332	49 (55.1)	48 (53.9)	0.876
Medications † ‡						
Steroid use	57 (60.6)	162 (47.5)	0.024	52 (58.4)	52 (58.4)	>0.999
Benzodiazepine	9 (9.6)	73 (21.4)	0.009	9 (10.1)	8 (9)	0.782
Opioids	59 (62.8)	233 (68.3)	0.309	55 (61.8)	57 (64.0)	0.746
Propofol	5 (5.3)	17 (5)	0.896	5 (5.6)	2 (2.3)	0.453
Delirium at the time of initial ICU admission	36 (38.3)	87 (25.5)	0.015	32 (36)	30 (33.7)	0.715

* Patients who met new Sepsis-3 definition. † Use of mechanical ventilation within 24 h after ED presentation. ‡ Medications administered to the patients prior to the diagnosis of delirium. APACHE, Acute Physiology and Chronic Health Evaluation; SOFA, Sequential Organ Failure Assessment; ICU, intensive care unit. The data are presented as mean ± standard deviations, median (IQRs) or numbers (%).

**Table 2:** Primary and secondary outcomes.

Outcomes	Before Matching					After Matching				
	Total (*n* = 435)	Treatment (*n* = 94)	Control (*n* = 341)	*p*	Effect Size	Total (*n* = 178)	Treatment (*n* = 89)	Control (*n* = 89)	*p*	Effect Size
Delirium-free days	13 (7–14)	11 (4–14)	13 (7–14)	0.042	0.188	11 (6–14)	11 (5–14)	12 (6–14)	0.894	0.035
Delirium-coma-free days	12 (4–14)	13 (6–14)	11 (2–14)	0.059	0.175	11 (4–14)	11 (3–14)	12 (4–14)	0.940	0.037
Incidence of delirium	256 (58.9)	62 (66.0)	194 (56.9)	0.113	0.141	111 (62.4)	57 (64.0)	54 (60.7)	0.622	0.046
Duration of delirium, days	1 (0–4)	2 (0–6)	1 (0–3)	0.032	0.196	1 (0–4)	1 (0–5)	1 (0–4)	0.604	0.082
Hospital LOS, days	14 (7–26)	16 (8–27.5)	13 (7–24)	0.065	0.147	15 (8–27)	16 (8–27)	13 (7–28)	0.693	0.117
ICU LOS, days	4 (3–7)	4 (3–8)	4 (3–7)	0.057	0.182	4 (3–7)	4 (3–7)	4 (3–7)	0.335	0.191
28-day mortality	95 (21.8)	23 (24.5)	72 (21.1)	0.486	0.053	37 (20.8)	21 (23.6)	16 (18.0)	0.336	0.111

The data are presented as median (IQRs) or numbers (%). ICU, intensive care unit; LOS, length of stay.

## Supplementary Materials

The following are available online at https://www.mdpi.com/2077-0383/9/1/193/s1: Table S1: Baseline characteristics of patients who met the new criteria for septic shock, Table S2: Outcomes of patients in septic shock who met the sepsis-3 criteria.
