# Supplementary material for: Impact of Vitamin C and Thiamine Administration on Delirium-Free Days in Patients with Septic Shock
Source: J Clin Med. 2020 Jan 10;9(1):193. doi: 10.3390/jcm9010193 (PMC7019730; doi:10.3390/jcm9010193)
Supplement: Supplementary file 1 [file jcm-09-00193-s001.pdf]

SUPPLEMENTARY MATERIALS

TABLE S1. Baseline characteristics of patients who met the new criteria for septic shock.

Variables	Before matching			After matching		
	**Treatment (*n* = 80)**	**Control (*n* = 215)**	***P***	**Treatmen t (*n* = 73)**	**Control (*n* = 73)**	***P***
**Age, years**	68 (60–74.5)	68 (60–76)	0.842	68 (61–74)	69 (59–76)	0.743
**Sex, male**	47 (58.8)	147 (68.4)	0.122	42 (57.5)	46 (63.0)	0.433
**Comorbidities**						
Diabetes	30 (37.5)	79 (36.7)	0.905	25 (34.3)	28 (38.4)	0.631
Hypertension	30 (37.5)	81 (37.7)	0.978	27 (37)	24 (32.9)	0.576
Cardiac disease	6 (7.5)	30 (14)	0.132	6 (8.2)	11 (15.1)	0.197
Cerebral vascular disease	7 (8.8)	17 (7.9)	0.814	6 (8.2)	7 (9.6)	0.782
Chronic lung disease	5 (6.25)	18 (8.4)	0.546	5 (6.9)	6 (8.2)	0.763
Chronic renal disease	5 (6.3)	17 (7.9)	0.630	7 (9.6)	12 (8.2)	0.564
Chronic liver disease	14 (17.5)	29 (13.5)	0.385	13 (17.8)	7 (9.6)	0.134
Hematologic malignancy	12 (15)	21 (9.8)	0.205	11 (15.1)	9 (12.3)	0.655
Metastatic cancer	16 (20)	59 (27.9)	0.192	15 (20.6)	22 (30.1)	0.194
**Infection focus**			0.067			0.853
Respiratory	21 (26.3)	81 (37.7)		21 (28.8)	22 (30.1)	
Non-respiratory	59 (73.7)	134 (62.3)		52 (71.2)	51 (69.9)	
**APACHE II**	31.2±8.2	30.3±9.5	0.281	30.8±8.2	31.6±10.0	0.619
**SOFA score**	12.2 (3.3)	11.3 (3.5)	0.015	11.9 (3.3)	12 (3.4)	0.913
**Laboratory tests**						
Lactate (mmol/L)	5.9±3.2	5.3±3.4	0.059	5.7±3.1	5.8±3.7	0.775
Albumin (mg/dl)	3.1±0.7	3.1±0.6	0.996	3.1±0.7	3.1±0.6	0.602
Creatinine (mg/dl)	2.1±1.5	1.9±1.8	0.132	2.0±1.5	1.9±1.2	0.763
**Mechanical ventilation use***	51 (63.8)	116 (54)	0.131	44(60.3)	46 (63)	0.724
**Medications†**						
Steroid use	54 (67.5)	124 (57.7)	0.125	47(64.4)	48 (65.8)	0.853
Sedation	63 (78.8)	169 (78.6)	0.978	58 (79.5)	58 (79.5)	>0.999
Benzodiazepine	8 (10)	52 (24.2)	0.007	8 (11)	8 (11)	>0.999
Opioids	55 (68.8)	151 (70.2)	0.805	50(68.5)	54 (74)	0.465
Propofol	5 (6.3)	13 (6.1)	>0.999	3 (4.1)	3 (4.1)	>0.999
**Delirium at the time of initial ICU admission**	51 (63.8)	116 (54)	0.131	44 (60.3)	46 (63)	0.724

*Use of mechanical ventilation within 24 hours after ED presentation. †Medications administered to the patients prior to the diagnosis of delirium. APACHE, Acute Physiology and Chronic Health Evaluation; SOFA, Sequential Organ Failure Assessment; ICU, intensive care unit. The data are presented as mean ± standard deviations, median (IQRs) or numbers (%).

TABLE S2. Outcomes of patients in septic shock who met the sepsis-3 criteria.

Outcomes	Before matching					After matching				
	**Total (*n* = 295)**	**Treatment (*n* = 80)**	**Control (*n* = 215)**	***P***	**Effect Size**	**Total (*n* = 146)**	**Treatment (*n* = 73)**	**Control (*n* = 73)**	***P***	**Effect Size**
Delirium-free days	12 (4–14)	10.5 (4–14)	12 (5–14)	0.171	0.156	11 (4–14)	11 (4–14)	11 (2–14)	0.531	0.109
Delirium-coma-free days	11 (2–14)	10 (1–-14)	12 (2–14)	0.208	0.142	11 (0–14)	11 (2–14)	11 (0–14)	0.587	0.097
Incidence of delirium	186 (63.1)	54 (67.5)	132 (61.4)	0.334	0.001	98 (67.1)	47 (64.4)	51 (69.9)	0.465	0.088
Duration of delirium, days	1 (0–4)	2 (0–6.5)	1 (0–4)	0.048	0.222	3.7 (5.6)	3.8 (5)	3.5 (6.2)	0.459	0.046
Hospital LOS, days	14 (7–26)	16 (8–27.5)	13 (7–24)	0.065	0.216	14.5 (8–27)	16 (8–27)	13 (8–24)	0.232	0.179
ICU LOS, days	4 (3–8)	5 (3–10.5)	4 (3–7)	0.044	0.234	4 (3–8)	4 (3–10)	4 (3–8)	0.539	0.209
28-day mortality	76 (25.8)	21 (26.3)	55 (25.6)	0.907	0.099	41 (28.1)	18 (24.7)	23 (31.5)	0.369	0.122

The data are presented as median (IQRs) or numbers (%). ICU, intensive care unit; LOS, length of stay.
